# Impacts of a Multi-Professional Family versus Isolated Intervention on Food Level Processing in Overweight Adolescents: A Randomized Trial

**DOI:** 10.3390/nu15040935

**Published:** 2023-02-13

**Authors:** Déborah C. S. Marques, Willian C. Ferreira, Isabella C. Santos, Joed J. Ryal, Marilene G. S. Marques, Fabiano M. Oliveira, Rute G. Milani, Jorge Mota, Pablo Valdés-Badilla, Braulio H. M. Branco

**Affiliations:** 1 Postgraduate Program in Health Promotion, Cesumar University, Maringá 87050-390, Brazil; 2 Interdisciplinary Laboratory of Intervention in Health Promotion, Cesumar Institute of Science, Maringá 87050-390, Brazil; 3 Medicine Course, Department of Health Sciences, Cesumar University, Maringá 87050-390, Brazil; 4 Research Centre of Physical Activity, Health and Leisure, Faculty of Sports, Laboratory for Integrative and Translational Research in Population Health (ITR), University of Porto, 4200-450 Porto, Portugal; 5 Department of Physical Activity Sciences, Faculty of Education Sciences, Universidad Católica del Maule, Talca 3530000, Chile; 6 Sports Coach Career, School of Education, Universidad Viña del Mar, Viña del Mar 2520000, Chile

**Keywords:** diet, food and nutrition, food quality, healthy foods availability, primary health care

## Abstract

The food consumption of adolescents has changed nowadays, with an increase in ultra-processed food that in general shows higher calories and lower nutrients. Because of this, the objective of this study was to investigate the impacts of a 12-week multi-professional family versus isolated intervention on the food level processing of overweight adolescents. A randomized clinical trial study was carried out in which adolescents (*n =* 43; mean aged 13.73 years) who were divided into FG—family group (*n =* 21; the adolescents performed the activities with their parents) and IG—isolated group (*n =* 22; the adolescents performed the activities alone). The parameters measured before and after 12 weeks of multi-professional intervention (physical exercise, nutrition and psychoeducation) were: body mass, height and body mass index-BMI. The level of food processing was analyzed using a three-day food recall (24hR), classified according to the Food Guide for the Brazilian Population (fresh, minimally, processed and ultra-processed foods). The main results show that there was only a significant reduction in the consumption of processed foods (FG: 7.93%; IG: 49.73%) and ultra-processed foods (FG: 35.06%; IG: 67.16%) in grams (FG: 22.29%; IG: 65.23%) and calories (*p <* 0.05; for all comparisons). The consumption of fresh foods in grams (FG:61.97%; IG: 147.13%) and calories (FG: 147.13%; IG: 118.03%) and minimally processed foods (FG: 27.45%; IG: 14.64%) in grams increased significantly (*p <* 0.05; for all comparisons). However, no significant differences were observed between all variables analyzed for the groups, nor any interaction (*p >* 0.05). In conclusion, both groups who participated in the activities showed positive changes with increased consumption of fresh foods and reduced consumption of processed foods, without difference between them.

## 1. Introduction

In recent years, there has been an increase in the consumption of processed foods in the Brazilian context, directly related to a nutritional transition leading to changes in dietary pattern, towards diets of low nutritional quality [1]. With regard to this, eating habits can be motivated by family, community, society, friends and social media advertisements [2]. Thus, these influences determine the food preferences of young people and, consequently, the quality of their diet [2]. Adolescence is marked by autonomy and independence in daily activities, with the adoption of new behaviors and experiences [3]. Therefore, the environment (family, colleagues, school, culture, relationship and beliefs, among others) in which the adolescent lives daily underlies their respective health habits [3]. However, established behaviors are shaped at a young age and maintained over time.

Therefore, understanding the trajectories of diet quality and identifying which influences are directly related to negative eating behaviors are necessary to positively modify health conditions [4]. In 2014, the Brazilian government published the second edition of the *Food Guide for the Brazilian Population* [5], which deals with a qualitative classification of foods (fresh products, minimally processed, processed and ultra-processed). In this sense, fresh products and minimally processed foods are those that have not undergone any changes after leaving nature (as fruits and salads) and have not had salt, sugar and oils added to their respective composition. In this way, the structure of the original food is unchanged [5]. On the other hand, processed and ultra-processed foods are formulated with excess sugar, salt and saturated or trans fats, in addition to added artificial ingredients [6]. The consumption of foods with a higher level of processing, i.e., processed and ultra-processed foods, according to the classification established by the Brazilian government [5], is correlated with excessive body mass gain, nutritional deficiencies and non-communicable chronic diseases (NCD) [7].

The demand for processed foods requires early lifestyle changes, with increased physical activity and improved dietary patterns. Thus, multi-professional actions are necessary to reverse this situation [6], since rapid weight gain in this age group is related to an increase in fat mass, accentuating comorbidities directly or indirectly associated with overweight and obesity [8]. Not only multi-professional actions but the participation of family members in the act of eating can influence lower-risk behaviors, such as an increase in the consumption of healthy foods and a possible decrease in the consumption of industrialized foods, in addition to lower consequences related to the use of illegal and alcoholic substances [9]. Because of this, the belief is that family involvement becomes a positive strategy in obesity treatment in adolescents. However, despite the family exerting a primary influence on the formation of eating habits, friends or colleagues in social life can directly influence the choices of children and adolescents [10].

Given this, understanding parental patterns and a possible relationship between food consumption in parents and adolescents become relevant to promote assertive interventions to combat overweight and obesity A recent study developed in Spain identified that nuclear families could promote better information about healthy eating habits for children and adolescents [11]. However, even the parents can only help the adolescents’ choices along with the individual’s internal desire to change eating behavior [12]. Thus, to the author’s knowledge, there is a gap in food processing studies in treating obesity in adolescents comparing isolated (IG) versus family groups (FG). Understanding the approach and identifying the best treatment method becomes important, since this nutritional transition negatively impacts adolescents’ body composition, causing health problems [1]. Therefore, the present study aimed to investigate the impacts of a family versus isolated multi-professional intervention on the food processing level of overweight adolescents.

## 2. Materials and Methods

### 2.1. Study Design

This study is a randomized, parallel-group, two-arm clinical trial with pre- and post-participation assessments over 12 weeks of intervention. 

As inclusion criteria, the following adolescents were recruited: (1) adolescents aged 12 to 17 years; (2) overweight or obese; (3) consent form signed by the responsible/guardian and adolescent; (4) availability to participate in multidisciplinary interventions three times a week for 12 weeks; and (5) attend the initial evaluations of the project. As exclusion criteria, adolescents: (1) did not complete any of the questionnaires requested; (2) presented orthopedic, cardiovascular and cognitive deficits that prevented the practice of physical exercise; (3) were on some type of restrictive diet (low-carb, low-fat or hypocaloric) or nutritional guidance during the 12 weeks of the study; (4) were using a psychotropic drug or appetite regulator; and (5) accidents that made it impossible to participate in practical interventions. According to Jensen et al. [13], 15 adolescents per group would be necessary to present an *α =* 0.05 and *β =* 0.80 in obesity intervention models.

The adolescents were randomized into two groups: family (the adolescent participated with the father or mother or legal guardian, called FG) and isolated (only the adolescent performed the interventions, called IG). Additionally, the present study followed the Consolidated Standards of Reporting Trials (CONSORT) guidelines [14]. The Ethics and Local Research Committee approved the research project under number 4.913.453/2021. The research followed all the recommendations proposed in resolution 466/2012 of the Ministry of Health of the Brazilian government. After approval by the committee, the project was registered with the Brazilian Clinical Trials Registry Platform (ReBEC) under the number RBR-8fp63gm.

### 2.2. Participants

**Figure 1:**
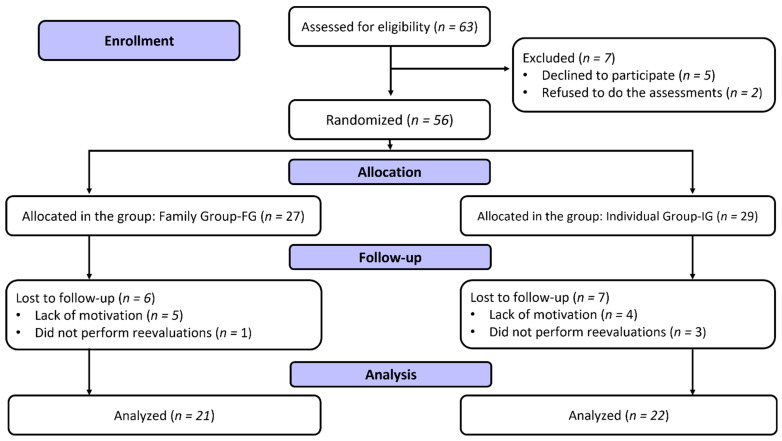
Study flowchart, based on CONSORT.

Sixty-three adolescents were eligible and randomized into two experimental groups, with 27 adolescents allocated to the FG and 29 to the IG. Therefore, 43 adolescents were analyzed at the end of the 12 weeks of intervention, 21 adolescents from the FG (male = 12 and female = 9) and 22 from the IG (male = 8 and female = 14), residing in a municipality in southern Brazil. Figure 1 presents the study flowchart.

### 2.3. Anthropometry

Height was measured using a Sanny^®^ brand stadiometer, Standard, following the standardization proposed by Lohman, Roche and Martorell [15]. Body mass (kg) was measured using a Welmy^®^ mechanical scale, with a capacity of 250 kg and an accuracy of 100 g. For the height and body mass data, the body mass index (BMI) was calculated by dividing weight by height squared (IMC = W/(H^2^)). The nutritional status of adolescents was classified according to the cutoff points established by the World Health Organization (WHO) [16], according to the percentiles: overweight were between the ≥85th and <95th percentile and those with obesity were ≥95th percentile. The BMI z-score was calculated in the World Health Organization computer application, according to the reference data of the WHO [16].

### 2.4. 24-Hour Food Record (24hR)

Habitual food consumption was assessed using a 24-h Food Recall (24hR). Participants filled in the 24hR at the beginning and end of the interventions in detail during two non-consecutive weekdays and one day at the weekend. The average ((day 1 + day 2 + day 3)/3) was used to establish the grams and kilocalories (kcals) of foods classified according to food processing [5,17]. Participants were asked to detail each food item, such as brand or restaurant names, and to label specific items (low-fat, 1% milk). The 24hR information was calculated using the Avanutri software (version 2004^®^, Avanutri Equipamentos de Avaliação Ltd.a, Três Rios, Rio de Janeiro, Brazil). The tables added foods unavailable in the program using the Brazilian Food Composition Table (TACO) [18], as suggested by Malta, Papini and Corrente [19].

### 2.5. Food-Level Processing

**Figure 2:**
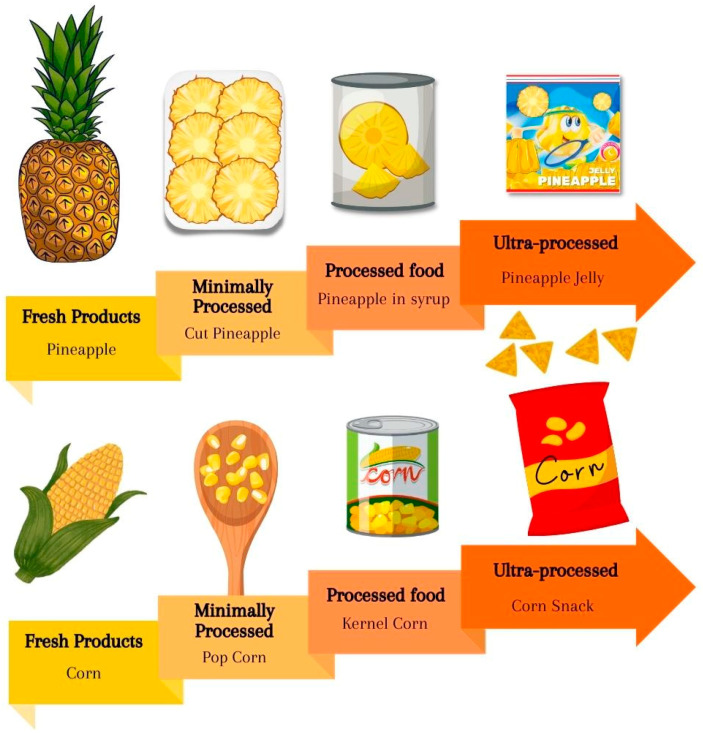
Didactic presentation of the level of food processing.

With the results found in 24hR, the amount in grams and kcals of each level of food processing was analyzed: Fresh Products, Minimally processed, Processed food, or Ultra-processed [20]. Fresh Products were obtained directly from plants, which have not undergone any alteration after leaving nature (such as fruits and salads). Minimally processed were subjected to minimal processes such as cleaning, removal of unwanted parts, packaging, drying, fermentation and other processes that do not add salt, sugar, or oils to the original food (such as fruit salad, orange juice). Processed food was produced from fresh food, but salt, sugar, oils and fats were added during its preparation (such as jams, cheese and bread). Ultra-processed foods had undergone industrial processes to be ready for consumption (such as stuffed cookies, candies, sweets, snacks, beverage drinks). The results were tabulated in an Excel spreadsheet and subsequently the pre- and post-intervention consumption were analyzed using the average of three days, conforming to the information described in the 24hR topic. Figure 2 shows a didactic presentation of the level of food processing. 

### 2.6. Tanner Scale

The Tanner Scale was used to monitor pubertal development [20], which systematized the sequence of pubertal events in both sexes through five stages, considering the female gender, breast development and the distribution and amount of hair: and in males, the appearance of the genitals and the amount and distribution of pubic hair [20].

### 2.7. Interventions

The interventions happen over 12 weeks, for three days a week. The meetings lasted for one and a half hours, along with the participation of the nutrition team once a week (30 min interventions each), psychology once a week (30 min interventions each) and physical education three times a week (lasting 1 h each). Interventions were previously designed and discussed among health professionals to determine a schedule and planning between areas. Thus, the contents were elaborated with common ground, focusing on the adolescent’s behavior change over the weeks. The only difference between the FG and the IG in the interventions was the family member’s participation in one of the groups. The protocol, time and interventions were the same for both groups. Additionally, the interventions were performed at different times so as not to interfere with the results.

Nutrition meetings were held in groups, focusing on changing eating behavior, with both groups receiving the same intervention. At all times, adolescents were encouraged to develop culinary skills in order to become independent and gain autonomy when choosing food preferences, thus encouraging the consumption of foods with a lower level of processing [5]. Group psychoeducation was carried out through theoretical-practical activities in which parents and adolescents were encouraged to participate in conversations. The central subjects were developed and devised according to the values and principles of the National Policy for Health Promotion (NPHP) [5], with cognitive and behavioral strategies to promote symptom reduction and food normalization, including self-monitoring, examinations of factors that stimulate binge eating, elaboration of an exercise program and prevention of situations involving binge eating [2,21].

The training sessions were developed in a circuit format, divided into two mesocycles (each with six weeks of duration), with training series A and B (divided into exercises with body mass, equipment and accessories, prioritizing the large muscle groups). There was no repetition count in the physical exercises but active and passive time were controlled via effort and rest ratio. In the first mesocycle, the effort:rest ratio was 30” by 30”, while, in the second mesocycle, the effort:rest ratio was 40” by 20”, using a 1:1 ratio between concentric and eccentric contractions.

### 2.8. Data Analysis

After confirming the normality of the data using the Kolmogorov-Smirnov test, the data were presented as the mean and standard deviation. To compare the pre- and post-intervention responses, a two-way analysis of variance (ANOVA) was performed, applying the Bonferroni post-hoc test (only if a significant difference is identified), assuming a *p <* 0.05. The greenhouse-Geisser correction was applied, if necessary, as well as Mauchly’s test of sphericity to test for equality between levels of independent variables. The deltas (post-intervention minus pre-intervention values) were also calculated. The effect size was calculated via Cohen’s *d* (1988): 0.20 (small effect), 0.50 (moderate effect) and 0.80 (large effect). All statistical analyses were performed using the Statistica package (Version 12.0, Stasoft, United States of America).

## 3. Results

A total of 43 adolescents (23 female and 20 male) were evaluated; 21 participants (9 female and 12 male) were allocated to the FG and 22 participants (14 female and 8 male) to the IG. Considering the Tanner questionnaire, the difference observed was the increase in the stage of sexual maturation during the 12 weeks of the intervention. However, this result was already expected, given that the adolescents were experiencing a period of growth and body complexion (*p =* 0.03). The information on age, body mass and height is presented in Table 1.

No group, time, or interaction effects were identified for body mass, BMI and BMI Z-score (*p >* 0.05). A time effect was identified for height, with higher values after the intervention period (*p =* 0.008; *d =* 0.06; small effect). Age showed a time effect, with high values after 12 weeks (*p* = 0.01; *d* = 0.10; small effect). The comparison of the level of processing of foods (fresh products, minimally processed, processed and ultra-processed) in grams and kcals is presented in Table 2.

**Figure 3:**
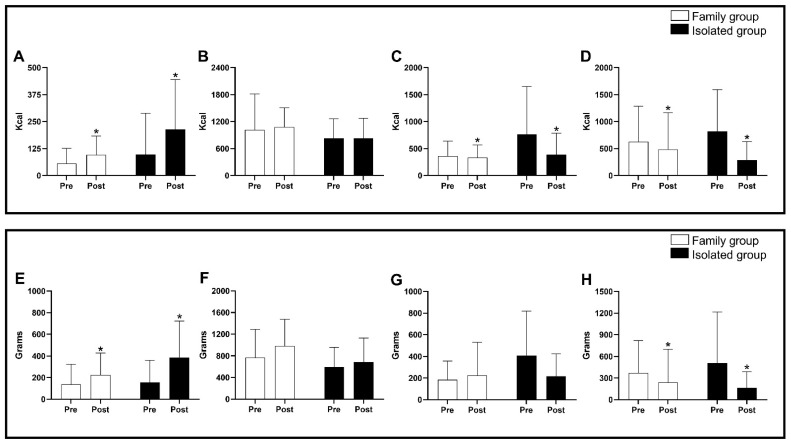
Consumption in grams and calories of fresh products, minimally processed, processed and ultra-processed foods before and after the intervention. Note: Data are expressed as a mean and standard deviation; * = difference in time before and after intervention (*p <* 0.05); Panel (**A**) = fresh foods in kcals; Panel (**B**) = minimally processed foods in kcals; Panel (**C**) = processed foods in kcals; Panel (**D**) = ultra-processed foods in kcals; Panel (**E**) = fresh food in grams; Panel (**F**) = minimally processed foods in grams; Panel (**G**) = processed foods in grams; Panel (**H**) = ultra-processed foods in grams.

Figure 3 presents the consumption in grams and calories of fresh products, minimally processed, processed, and ultra-processed foods before and after the intervention.

For the consumption of fresh products in kcals, only a time effect was observed with an increase in the consumption of these products after the intervention (*p =* 0.005; *d =* 0.55; moderate effect). For the consumption of fresh products in grams, there was only a time effect with an increase in the consumption of these products after the intervention (*p* = 0.0006; *d* = 0.84; large effect). For the consumption of minimally processed foods in kcals, there were no group, time, or interaction effects (*p >* 0.05). However, no significant differences were observed for the consumption of minimally processed foods in kcals and grams (*p >* 0.05). The consumption of processed foods in kcals was only a time effect with reduced consumption after the intervention (*p =* 0.04; *d =* 0.31; moderate effect). There were no group effects for consuming processed foods in grams at any time and no interaction (*p* > 0.05). For the consumption of ultra-processed foods in kcals, there was a time effect with a reduction in the consumption of these products after the intervention period (*p* = 0.01; *d* = 0.48; moderate effect). For the consumption of ultra-processed foods in grams, there was also a time effect with a reduction in consumption of these products after the intervention period (*p* = 0.01; *d* = 0.40; moderate effect). Finally, no significant differences were observed among groups or interactions in any of the investigated variables, i.e., fresh food, minimally processed, processed, or ultra-processed foods, with *p >* 0.05. 

## 4. Discussion

Considering the main aim of the present study, which was to investigate the impacts of 12 weeks of family versus isolated multi-professional intervention on the diet of overweight adolescents, it was observed that both isolated and family participation showed a significant improvement in the level of food processing after the intervention period, i.e., both were effective in increasing the food quality of the adolescents participating in the present study.

The main results found in the study were: (i) a time effect, with an increase in grams and calories in the consumption of fresh products; (ii) a time effect, with an increase in consumption in grams of minimally processed foods; (iii) a time effect, with reduced calorie consumption from processed foods; and (iv) a time effect, with a decrease in grams of ultra-processed foods, with all differences observed in the post-intervention period.

On the other hand, significant differences in body mass, BMI, BMI z-score, consumption in kcals of fresh foods and consumption in grams of processed foods were not found. When investigating the intergroup differences, these differences were not found either. The results suggest that, regardless of family involvement, both groups showed positive results regardless of parental or guardian involvement.

Family support can be complex, so support does not only imply encouraging participation together in activities but also changing the family’s habits at home [22]. Incentives should be given when purchasing fruits, legumes and vegetables, avoiding the purchase of processed foods, reducing the frequency of consumption of fast-food and presenting positive examples of food and assertive behaviors regarding food consumption [6]. Assigning multi-professional interventional strategies with family encouragement and support for physical activity tends to provide an environment conducive to behavior change [23]. Therefore, the conclusion was that this study positively influenced both groups to change their eating behavior towards daily healthy habits.

The progressive gain of autonomy during adolescence makes the individual susceptible to making good or not-good food choices regarding nutritional quality [23]. These choices influence the nutritional status, causing weight gain in this age group [8]. Therefore, multi-professional interventional actions can help with the prevention and progression of some complications associated with excess weight [6]. The complexity of achieving changes in behavior and adherence to healthy eating habits requires the action of different health professionals to adopt effective strategies and make the recovery process from health conditions more effective [17,21,24].

In line with the present study, the literature presents similar results when evaluating the same variables of body mass, BMI and z-score [9]. The study developed by Branco et al. [21], with 12 weeks of intervention, also did not identify significant reductions in body mass and BMI in two contrasting groups that underwent different physical activity practices (weight training or “functional” training). However, the treatment of obesity does not only aim at actions focused on weight loss and its related indicators, such as body mass and BMI; in this sense, other aspects should be analyzed, such as body composition and food quality [25]. Analyzing only the anthropometric variables (body mass and BMI) in the weight loss process is not enough to verify positive health outcomes; in this process, increase in lean mass may occur concomitantly with a reduction in fat mass but without showing significant differences in body mass [21].

The consumption increases of fresh products and minimally processed foods, as well as the reduction of processed and ultra-processed, suggest that theoretical-practical interventions promoted an improvement in the food quality of adolescents; some parameters imply the promotion of healthy autonomy in food decisions and adherence to healthy behaviors. Recent evidence reinforces the need to increase the consumption of fresh and minimally processed foods, reducing the intake of processed and ultra-processed foods [26] to avoid obesity in adolescents [27].

The consistent improvement in these aspects, especially in adolescence, helps to prevent the manifestation of cardiovascular diseases [28]. Processed foods show worse nutritional quality due to the exacerbated amount of sugar, sodium and trans and saturated fats [6]. Excessive consumption of these elements is related to increased mortality risk from cardiovascular diseases and NCD [6]. Therefore, food improvement quality contributes to energy reduction density and can help the weight loss process [28].

The adolescents in the present study participated in the activities during the COVID-19 pandemic with many municipal and state restrictions (between March to August/21). In this scenery, caregivers and adolescents had to adapt to the digital environment and experienced cases in which they were overloaded with household chores and work at home, influencing the search for ready-to-eat and quick-to-prepare food [29,30]. These attitudes impacted weight gain and body composition [31], as lifestyle changes result in increased energy intake and decreased caloric expenditure [15].

However, the intake influence of certain foods is shaped by the availability at home and, because of this, the parenting style is directly reflected on the acquisition of certain behaviors [32]. Moderate restriction by caregivers is considered beneficial since moderately restricting adolescent behaviors is associated with more fruits, less fatty foods, fewer sweets and fewer calories than severe restriction [15]. However, severely restricting food is related to the desire to eat compulsively, even without hunger [32]. Because of this, the pressure exerted on food can cause negative behaviors; thus, reducing excessive control in adolescents’ eating attitudes can be established as the best method for changing eating behavior [32].

Thus, specific resources are necessary to adopt healthy eating habits, physical activities and emotional control [30]. With adverse emotional effects caused by COVID-19 of anxiety, sadness, anger and loneliness [30], adolescents lacked actions aimed at their emotional and binge eating. Even though the influence of children’s eating behaviors varies according to age, childhood is attributed as the period of dependence on family members, influenced by the home environment [32]. The family is the support to sustain the young person’s involvement in the treatment of obesity [2].

However, even if the family context can determine the environment, the transformation process requires the adolescent’s will and involvement [17]. The study presents similar responses to Oliveira et al. [17] with significant improvement in eating behavior after 12 weeks of multi-professional interventions to combat obesity in adolescents. The present study brings relevant aspects to the clinical design of interventions focused on improving the dietary pattern of adolescents. Regardless of the treatment method, with or without family involvement, it is suggested that adolescents be consulted, considering their preferences and expectations for treatment. In this way, the belief is that the adolescent will be able to feel more comfortable, with greater adherence to the proposal to improve health indicators made by the multi-professional team.

The answer to this question becomes broader since the multi-professional team will need to evaluate the profile of each adolescent and identify which intervention (family or isolated) can generate more adherence in changing eating behavior and encouraging physical activity. Family involvement can often be challenging and stressful due to demands, pressure, the shame of exposure to treatments, guilt, or even self-criticism, for example [15]. Often, the maternal stimulus to perform restrictive diets with a focus on weight loss can be contextualized as a predictor of exaggeration and binge eating; therefore, the individual participation of adolescents may be more advisable in most clinical cases [15]. Considering the aspects listed, health professionals who care for overweight adolescents must recognize the different dysfunctional behaviors and contexts in order to provide an environment conducive to change. Public policies must guide change by integrating multi-professional teams, schools, families, communities and media in providing a healthy lifestyle [17,21].

Four limitations may be highlighted of this study: (i) not analyzing the power supply in the middle of the study to advise on possible irregularities; (ii) control of eating after one month of this study to verify whether the change in eating habits was persistent; (iii) a lack of other anthropometric measures, such as waist circumference, hip circumference, neck circumference, arm circumference, as well as body composition measurements (lean mass, fat mass and body fat percentage), as the body composition variables are positively correlated with BMI in adolescents [33]; and (iv) 24hR is an instrument that may present some variability even with familiarization. Therefore, new studies with more accurate measures to weigh the food and calculate macronutrients, micronutrients, kcals and food level processing could be relevant to identify possible differences between parents or individual interventions for adolescents with overweight or obesity. Despite this, the results were in line with the studies by Branco et al. [21,34,35], showing good reliability for application of the multi-professional intervention model.

## 5. Conclusions

The analysis of the benefits of healthy eating should not be limited to weight status or BMI. The positive eating changes need to transcend numerical values and could focus on food quality and long-term responses. The changes in the dietary profile of overweight adolescents showed significantly positive responses, with a consumption reduction of processed and ultra-processed foods in grams and kcals, besides an increase in fresh foods in grams and kcals. However, when comparing the FG with the IG, there were no significant differences in the consumption in grams and kcals of fresh products after the multi-professional interventions. Finally, the best strategy to be followed to provide positive changes in the diet of overweight adolescents will depend on preference, family relationship and behavior.

## Figures and Tables

**Table 1:** Mean values, standard deviation, absolute delta and Cohen’s *d* of general characteristics and anthropometry of adolescents before and after the intervention.

Variables	FG		Delta	Cohen’s *d*	IG		Delta	Cohen’s *d*
	Pre	Post			Pre	Post		
Age (years old) *	14.24 ± 2.61	14.52 ± 2.62	0.29 ± 0.46	0.11	13.23 ± 2.27	13.45 ± 2.39	0.23 ± 0.43	0.10
Body Mass (kg)	76.95 ± 22.30	77.47 ± 21.79	0.52 ± 2.12	0.02	83.52 ± 28.76	83.22 ± 26.60	−0.30 ± 3.01	−0.01
Height (m^2^) *	1.62 ± 0.13	1.63 ± 0.13	0.01 ± 0.01	0.07	1.64 ± 0.10	1.65 ± 0.10	0.01 ± 0.01	0.06
BMI (kg/m^2^)	28.89 ± 6.11	28.77 ± 5.78	−0.12 ± 0.98	−0.02	30.77 ± 9.47	30.52 ± 8.84	−0.25 ± 1.11	−0.03
BMI Z-score	2.17 ± 1.36	2.12 ± 1.23	−0.05 ± 0.26	−0.04	2.52 ± 1.63	2.45 ± 1.53	−0.07 ± 0.20	−0.05

Note: Data are expressed as mean and standard deviation (±); FB = family group; IG = isolated group; BMI = body mass index; Pre = pre-intervention; Post = Post-intervention; * = time difference, pre-different from post-intervention (*p <* 0.05).

**Table 2:** Values of the mean and standard deviation of the absolute delta, *p-*value and *d* Cohen’s *d* of kcals and kilograms of fresh products, minimally processed, processed and ultra-processed foods of adolescents.

	Kilocalories				Kilograms			
Processing Level	Δ Absolute FG (Mean ± SD)	Δ Absolute IG (Mean ± SD)	*p-*Value	Cohen’s *d*	Δ Absolute FG (Mean ± SD)	Δ Absolute IG (Mean ± SD)	*p-*Value	Cohen’s *d*
Fresh Products	40.2 ± 103.1	116.0 ± 224.0	*p =* 0.16	0.43	86.5 ± 274.4	231.1 ± 285.8	*p =* 0.09	0.51
Minimally Processed	58.9 ± 784.6	4.37 ± 574.5	*p =* 0.79	0.02	210.4 ± 639.8	87.5 ± 515.8	*p =* 0.49	0.21
Processed	−28.9 ± 327.7	−382.2 ± 833.5	*p =* 0.07	0.55	39.9 ± 347.0	−192.2 ± 465.2	*p =* 0.07	0.56
Ultra-processed	−139.5 ± 854.1	−537.1 ± 828.0	*p =* 0.12	0.47	−130.2 ± 510.1	−341.5 ± 742.5	*p =* 0.28	0.33

Note: Data are expressed as mean and standard deviation, SD (±); FG = family group; IG = isolated group; Δ = delta; *p >* 0.05 for all comparisons.

## Data Availability

The data generated during the study will be informed when requested.
